# Surgery for Colorectal Cancer Associated with Crohn’s Disease—Toward a Medical Treatment Strategy Based on the Differences Between Japan and Western Countries

**DOI:** 10.3390/cancers17050860

**Published:** 2025-03-03

**Authors:** Yuki Sekido, Takayuki Ogino, Mitsunobu Takeda, Tsuyoshi Hata, Atsushi Hamabe, Norikatsu Miyoshi, Mamoru Uemura, Tsunekazu Mizushima, Yuichiro Doki, Hidetoshi Eguchi

**Affiliations:** 1 Department of Gastroenterological Surgery, Osaka University, Osaka 565-0871, Japan; togino04@gesurg.med.osaka-u.ac.jp (T.O.); mtakeda@gesurg.med.osaka-u.ac.jp (M.T.); tsuyoshihata@gesurg.med.osaka-u.ac.jp (T.H.); ahamabe@gesurg.med.osaka-u.ac.jp (A.H.); nmiyoshi@gesurg.med.osaka-u.ac.jp (N.M.); muemura@gesurg.med.osaka-u.ac.jp (M.U.); ydoki@gesurg.med.osaka-u.ac.jp (Y.D.); heguchi@gesurg.med.osaka-u.ac.jp (H.E.); 2 Department of Colorectal Surgery, Dokkyo Medical University, Tochigi 321-0293, Japan; t-mizushima123@dokkyomed.ac.jp

**Keywords:** Crohn’s disease, Crohn’s disease-associated cancer, anal and rectal cancer

## Abstract

With advances in the treatment of Crohn’s disease (CD), the number of long-term cases is increasing, along with the incidence of CD-related cancers. Here, we discuss the clinical features, diagnosis, treatment, prognosis, and surveillance of CD-related cancers. There are regional differences in the common sites and histological types of CD-related cancers, with right-sided colon cancer accounting for 40% of cases in Europe and the US, and squamous cell carcinoma being common. In Japan, rectal and anal cancers account for 80% of cases, and mucinous carcinoma is common. The prognosis of CD-associated colon cancer and sporadic colon cancer is the same; however, the prognosis of CD-associated rectal cancer is clearly worse than that of sporadic rectal cancer. Early diagnosis is important to improve the prognosis of CD-associated rectal cancer, and it is necessary to establish a surveillance method for CD-associated cancer that combines colonoscopy, anesthetic proctoscopy, and imaging, as appropriate. The basic treatment for CD-related cancer is surgical resection; however, the criteria for selecting the surgical procedure are unclear, and there is no clear evidence for multidisciplinary perioperative treatment including chemotherapy and radiotherapy. Additionally, CD-related rectal and anal cancers have a higher local recurrence rate than that of sporadic rectal cancers; therefore, thorough local control is important. Furthermore, CD-related cancers have different epidemiologies in different regions; therefore, unique diagnostic and treatment strategies must be established for each region.

## Simple Summary

With advancements in Crohn’s disease (CD) treatment, long-term cases and CD-related cancers are increasing. This article reviews the clinical features, diagnosis, treatment, prognosis, and surveillance of CD-related cancers. Regional differences exist in cancer types: in Europe and the US, 40% are right-sided colon cancers, while Japan sees 80% in rectal and anal cancers, with mucinous carcinoma being common. The prognosis for CD-associated colon cancer is similar to sporadic colon cancer, but CD-associated rectal cancer has a worse prognosis. Early diagnosis and a surveillance strategy combining colonoscopy, anesthetic proctoscopy, and imaging are critical. Surgical resection is the primary treatment, yet criteria for procedures and evidence for multidisciplinary perioperative care remain unclear. CD-related rectal and anal cancers have higher local recurrence rates, necessitating thorough local control. Regional variations in epidemiology highlight the need for tailored diagnostic and treatment strategies for CD-related cancers.

## 1. Introduction

Crohn’s disease (CD)-associated cancers are a major cause of death in patients with CD. Warren et al. first reported CD-associated cancer; they described a case of ascending colon mucinous carcinoma in 1948, 16 years after Crohn et al. established the concept of CD [1,2]. In Japan, the number of reports has increased since the 2000s and CD-related cancers have been recognized as an important complication of long-term CD. One reason for the increase in the number of neoplasms is thought to be that advances in drug therapy have greatly extended the period of intestinal preservation, and that the number of resection surgeries has decreased relatively, and it is thought that this will continue to increase in the future. The rectoanal region is the most frequently affected area in Japan. Although CD is associated with various inflammatory lesions, it is difficult to distinguish it from cancer; therefore, careful follow-up is required. In addition, although the terminal ileum is a common site for CD, there are few reports on small intestinal cancer, and there are still several unknown factors. Although small intestinal cancer is rare, in recent years, small intestinal endoscopy and capsule endoscopy have become more widespread, and the number of cases where a preoperative diagnosis can be obtained may increase in the future. In this article, we discuss the clinical features, diagnosis, treatment, prognosis, and surveillance of CD-related cancers.

## 2. Incidence and Risk Factors

According to a literature review, the incidence of CD-related cancers in Japan is 0.23%, with a median follow-up period of 12.55 years [3]. The risk of colorectal cancer in patients with CD is significantly higher than that in the general population, at 3.2 times [4]. In Europe and the United States, the relative risk of colorectal cancer in patients with CD is 2.5 times higher than that in the general population (4.5 times higher in patients with CD of the colon in particular); however, although the risk of colon cancer is higher in patients with CD, a higher risk of rectal cancer has not been shown [5,6]. Several risk factors of CD-related colorectal cancer are reported. The first is the number of years since the onset of CD: Similar to that in ulcerative colitis (UC), the risk of cancer increases with the number of years since diagnosis. The rate of cancerization is 2.9% after 10 years, 5.6% after 20 years, and 8.3% after 30 years [7]. The second is the age at CD diagnosis; according to one population-based study, CD cases diagnosed under the age of 30 have a 20.9 times higher risk of colorectal cancer than those diagnosed after the age of 30 [8]. The third factor is a family history of colorectal cancer; patients with CD with a family history of colorectal cancer have a 3.7 times higher risk than those without a family history [9]. Fourth, the extent of the affected area of the intestinal tract in CD and the risk of colorectal cancer in patients with CD with extensive colorectal lesions are extremely high (18.2 times) [10]. The presence of primary sclerosing cholangitis (PSC) is also a risk factor [11]. The risk of developing not only colorectal cancer but also small intestinal cancer is 33.2 times higher in people with CD than in the general population [12], and the risk of CD-related small intestinal cancer increases to 46 times in cases where the duration of CD is ≥8 years [13].

With regard to the risk of malignant disease due to treatment for Crohn’s disease, a prospective cohort study of IBD cases in Europe has shown that there is an increased risk of lymphoma due to thiopurines [14]. A retrospective cohort study of Japanese patients did not find an increased risk of lymphoma due to thioprines [15]. A prospective cohort study of CD patients in North America reported that infliximab monotherapy was not a risk factor for malignancies [16], but it is considered to be a risk factor for lymphoma when used in combination with thiopurine drugs [17]. There is no clear evidence at present for other biological agents either, but it is thought that the risk of malignancies should be kept in mind when deciding on the indication for use in combination with immunomodulators.

## 3. Clinical Features

Crohn’s disease is most common in young people in their late teens and 20s and causes chronic inflammation of the entire digestive tract, from the mouth to the anus. As inflammation progresses, it causes narrowing of the digestive tract, fistula formation, and bleeding. Pathologically, it is characterized by longitudinal ulcers in the lumen of the digestive tract, mainly on the mesenteric side, and noncontiguous intestinal inflammatory lesions (skip lesions). The chronic inflammatory environment of the digestive tract disrupts tissue homeostasis, promoting abnormal cell growth and the development of dysplasia in the intestinal mucosa, which can progress to cancer (colitis-associated cancer). As the number of long-term CD cases and patients with CD increases, the number of CD-related cancer cases in Japan is also increasing, which is becoming a problem.

Although the average age at diagnosis of sporadic colorectal cancer in Japan is 65 years, CD-associated cancer occurs at a younger age (47 years), and 66% of patients are men [18]. The location of CD-associated cancer differs by region and race. In Europe and the United States, right-sided colon cancer accounts for approximately 40% of all colon cancer cases, and the proportion of right-sided colon cancer is higher than that of sporadic colorectal cancer. In Japan, 80% of CD-related cancers occur in the rectoanal region (including anal fistula cancers) [3,18,19,20]. In addition, multiple colorectal cancers are found in >10% of the patients, and it is clearly more frequent than sporadic colorectal cancer [18,21]. Regional differences in tumor occurrence may be due to genetic, environmental, and lifestyle factors, and the analysis of these differences may provide important insights into cancer prevention.

**Figure 1:**
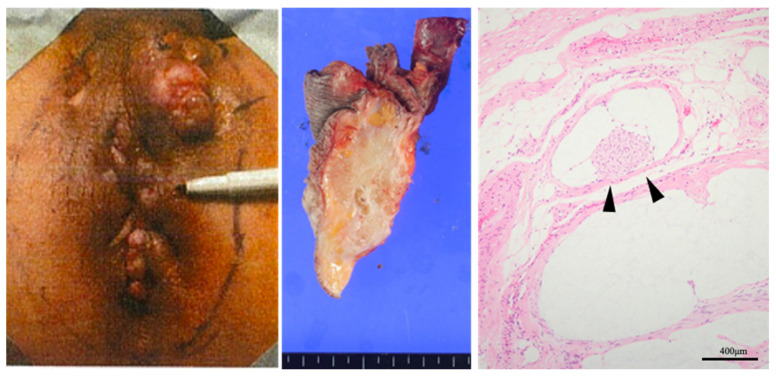
Gross and histopathological findings of CD-associated anorectal cancer. At the time of diagnosis, bloody mucus was observed in the anal region (**left**). In the cross-section of the specimen resected after neoadjuvant chemoradiotherapy, tumor tissue containing mucus was observed (**center**). Microscopic findings showed floating carcinomas indicated by the arrowheads within the mucinous nodules (**right**).

In terms of the macroscopic type of colorectal cancer, the ulcer-localized type accounts for approximately 60% of sporadic colorectal cancers in Japan; however, in CD-related cancer, 30% of colon cancers and half of rectal cancers are classified as unclassifiable, which is the most common macroscopic type [18]. In terms of histological type, >90% of sporadic colorectal cancers are either well-differentiated or moderately differentiated adenocarcinomas, whereas >40% of CD-associated colorectal cancers are mucinous carcinomas. Additionally, the proportions of signet ring cell carcinomas and poorly differentiated adenocarcinomas are also high [18], and this tendency was stronger with greater tumor depth of invasion [22]. A pathological image of a Japanese case of rectoanal mucinous carcinoma associated with CD is shown in Figure 1. Macroscopically, a scarred lesion with an ulcer in the anal canal was observed, making it difficult to determine the extent of the tumor. Histologically, the tumor formed a prominent mucinous lake. However, there are many reports of squamous cell carcinoma from Europe and America, and there are only a few reports from Asia; therefore, there are regional differences in histological types [3].

## 4. Diagnosis

Early diagnosis is extremely important for improving the prognosis of patients with CD-related cancer. Even when a high risk of CD-related cancer is recognized, it is often difficult to perform surveillance tests because of the narrowing of the digestive tract caused by CD, and many cases are diagnosed after cancer progression. According to a large-scale cohort study, 56% of the cases were diagnosed after a thorough examination of symptoms [21]. In the case of CD-related rectal and anal cancer, anal lesions of CD are accompanied by symptoms such as inflammation, pain, induration, stenosis, bleeding, anal pain, and mucus secretion; however, it is extremely difficult to distinguish these from cancer symptoms. In cases where symptoms worsen, such as worsening pain from anal lesions or an increase in mucus discharge, cancer should be suspected, and examination under anesthesia (EUA) should be considered. During EUA, the patient is placed in a position (such as the lithotomy position) that allows for sufficient observation, and a tissue biopsy is performed through the anus. Biopsy samples are taken from the perianal area, including the secondary orifice of the fistula, fistula tracts, and anal canal, and the pus and mucus are subjected to cytological examination. As malignant findings cannot be detected in many cases with a single biopsy, the examination should be repeated, and patients should be provided with sufficient explanations regarding the significance of the examination. In addition, in cases of small-bowel cancer, the small bowel often has multiple stenotic lesions, and in some cases, biopsies of the distal intestinal tract cannot be performed due to stenosis. In many cases, small bowel cancer is diagnosed based on intraoperative findings or postoperative pathological results; CD-related small bowel cancer is diagnosed after surgery in approximately 70% of cases, and preoperative diagnosis is still a challenge, especially with bowel stricture [23]. On the other hand, in recent years, double-balloon small intestinal endoscopes and capsule endoscopes have become available, and in cases where there is no risk of obstruction, these may lead to early detection.

**Figure 2:**
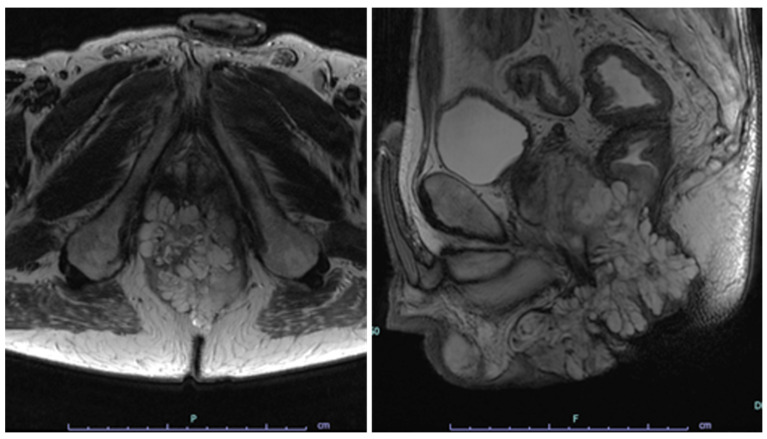
MRI T2-weighted images of a case of anorectal cancer, axial (**left**) and sagittal (**right**). A multilocular cystic lesion is seen in the rectum and anal canal.

Although colonoscopy is the standard method of examination, the examination itself cannot be performed in several cases because of anal stenosis or pain. In some cases, it is better to perform it at the same time as the aforementioned EUA. Magnetic resonance imaging (MRI) can identify abscesses, fistulas, and inflammatory changes and is useful for understanding the extent of lesions. It is also useful for diagnosing tumor invasion of other organs and determining the extent of resection. Typical MRI findings in CD-related rectal and anal cancers include high-signal areas resembling granulation tissue and colloid retention on T2-weighted images (Figure 2). Computed tomography (CT) is suitable for evaluating tumor lymph node metastasis, distant metastasis, and lymph node metastasis; however, it is not useful for early detection. The detection rate of mucinous carcinoma with fluorodeoxyglucose positron emission tomography (FDG-PET) is considered low [24], and because accumulation is observed in inflamed areas, it is difficult to distinguish between cancer and inflammatory lesions; therefore, the diagnostic usefulness of FDG-PET has not been established. Tumor marker tests are easy to perform, and CD-related cancers can be diagnosed with an increase in carcinoembryonic antigen levels; however, in many cases where marker levels rise, the cancer has already progressed; therefore, tumor markers are not suitable for early diagnosis. In some cases, only the carbohydrate antigen 19-9 or serial Tn antigen levels increased. It is often difficult to clearly distinguish between inflammation and tumors using the currently available testing methods; therefore, testing methods that can sensitively monitor inflammatory carcinogenesis need to be developed.

## 5. Treatment

Currently, the standard treatment for CD-related colorectal cancer is surgical resection. The principle of intestinal surgery for patients with CD without cancer is to minimize the extent of intestinal resection. However, in CD-related cancer surgery, no cancerous tissue must be retained on the surgical dissection surface. Unlike UC, where the extent of the affected area can be determined preoperatively, CD can have various inflammatory distributions. Therefore, the extent of resection should also consider synchronous multiple cancers and dysplasia, which occur in 4% and 30% of cases, respectively. Also, among colectomy cases, 14% have been reported to have developed metachronous multiple cancers within 4 years [25]. In some cases, total colectomy is performed for resecting synchronous multiple colorectal cancers and dysplasia that have not been diagnosed and preventing metachronous carcinogenesis. However, in other cases, partial resection is chosen, considering factors such as the length of the remaining intestinal tract and the acceptability of an artificial anus, and there are no clear standards for surgical procedures for CD-related colorectal cancer.

In Japan, many cases of rectal cancer are associated with rectal and anal lesions in CD, and rectal resection is often performed in such cases. However, if there is an invasion of surrounding organs, such as the prostate, bladder, uterus, or vagina, extended surgery, such as total pelvic exenteration, is chosen to ensure negative surgical margins. In practice, it is often difficult to determine the extent of perineal resection in cases where the anal canal is highly fibrotic or complicated by anal fistulae due to CD-related anal lesions or in cases where there is no preoperative diagnosis.

In some cases, preoperative chemoradiotherapy (CRT) is used for CD-related rectal and anal cancers. It has been used to improve the local control rates in sporadic rectal cancer through R0 resection. Chemoradiotherapy is safe for CD-related rectal and anal cancers, but there are no reports of improved survival or reduced recurrence rates [26,27,28]. The efficacy of chemotherapy and radiotherapy is lower for mucinous carcinoma, which is common in CD-related cancers, than for other histological types, and it is difficult to assess treatment efficacy [29]. However, there are cases in which the tumor-suppressing effect of CRT is observed, even in mucinous carcinoma [30], and further evidence is needed in this area. Although CRT is expected to be an effective strategy, CD may worsen due to CRT; therefore, in practice, individualized treatment will be necessary.

In patients with advanced or recurrent cancer with distant metastases, chemotherapy is often administered if the patient’s general condition is good. However, there are no chemotherapy regimens with evidence specific to CD-related cancers, and chemotherapy is currently administered according to the guidelines for the treatment of colorectal cancer. The risk of adverse gastrointestinal events is increased due to chronic inflammation, shortening of the residual intestinal tract due to multiple previous surgeries, and diarrhea due to colonic resection and artificial anus, etc.; therefore, chemotherapy for CD cases is often difficult to manage. In addition, chemotherapy is less effective on mucous membrane cancer, and it has a poorer prognosis than those of cancers of other tissue types [31]. In a study on the tolerability of chemotherapy for CD-related cancer, a multidrug regimen containing 5-FU was administered to five patients with small-bowel–large-bowel-type CD, and grade 3 diarrhea was observed in three cases, suggesting a link between the length of the remaining small intestine and diarrhea [32]. Regarding the relationship between CD disease activity and chemotherapy, chemotherapy itself is reported to have an immunosuppressive effect, which improves the control of CD [33]. Currently, there are no clear criteria for determining the appropriateness of chemotherapy, and decisions are based on the patient’s general condition and disease stage.

There are no evidence-based opinions or reports on the extent of lymph node dissection or chemotherapy during surgery for CD-related small intestinal cancer, and treatment is currently performed in the same manner as that for colorectal cancer. In small intestinal cancer, preoperative diagnosis is rare, and postoperative diagnosis is often based on a pathological examination of the resected specimens [23,34]. Therefore, while performing a surgical procedure that preserves a chronically inflamed intestinal tract, such as stenoplasty for CD intestinal strictures, it is desirable to perform an intraoperative biopsy of the same area. However, it is sometimes difficult to reach an accurate diagnosis through intraoperative rapid pathological diagnosis. In cases of intestinal obstruction symptoms that are completely unresponsive to medical treatment or where symptoms rapidly worsen after a long period of remission, the possibility of small intestinal cancer must be considered. Also, if the length of the remaining small intestine can be secured, regional lymph node dissection may be considered in parallel.

## 6. Prognosis

The stage at diagnosis does not differ between CD-associated colorectal cancer and sporadic colorectal cancer, but CD-associated colorectal cancer has a poor prognosis, with a hazard ratio at 1 and 5 years of 1.82 (95% CI: 1.36–2.43) and 1.57 (95% CI: 1.24–1.99), respectively [35]. A meta-analysis comparing the mortality rate of CD-related colorectal cancer with that of sporadic colorectal cancer found that the standardized mortality ratio was 1.34 (95% CI: 0.54–3.33), indicating that CD-related colorectal cancer has a higher risk of mortality [36].

**Figure 3:**
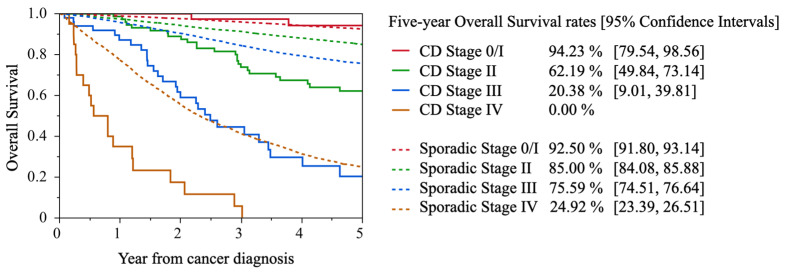
Five-year Overall Survival of CD-associated colorectal cancer and sporadic colorectal cancer patients by Stage. Modified citation from reference [18].

In Japan, the 5-year survival rate for all CD-related colorectal cancers is 54.0%, which is lower than the rate for all sporadic colorectal cancers (71.2%) [18,21]. The 5-year survival rate for stage 0/I was 94.2%, which was good, but the 5-year survival rates for Stage II and Stage III were 62.2% and 20.4%, which were clearly poor compared to those for sporadic colorectal cancer, and all patients with Stage IV cancer died within 5 years (Figure 3). Additionally, 81.5% of CD-related colorectal cancers were located in the rectum and anus; although the prognosis of CD-related colon cancer and sporadic colon cancer was the same, the prognosis of CD-related rectal and anal cancers was worse than that of sporadic rectal cancer. There was no significant difference in the recurrence rate between CD-related colon cancer and sporadic colon cancer. However, the recurrence rate for CD-associated rectal cancer was 39.6%, which was higher than that for sporadic rectal cancer (22.0%). The most common form of recurrence in CD-associated rectal cancer was local recurrence, which accounted for more than half of all recurrences [3].

The 5-year survival rate of CD-related small bowel cancer is approximately 30%, and it has a poor prognosis [25]. The majority of CD-related small bowel cancers occur in the ileum, and it is difficult to directly compare it with the location of sporadic small bowel cancers, which are more common in the duodenum and jejunum.

## 7. Surveillance

Western guidelines recommend regular colonoscopy for the surveillance of CD-related cancer. Surveillance for cancer should begin 8 years after the onset of the disease in cases where inflammation has spread to >30% of the colon and from the time of onset in cases where PSC is also present, regardless of the extent of the lesion [37,38]. As the number of cases of CD-associated small bowel cancer is limited, and it is often difficult to directly observe lesions, no surveillance method has been established at present. In Japan, where rectal and anal cancers account for the majority of cancer cases, unique cancer surveillance methods and treatment strategies are required.

Examination under anesthesia is practical for the examination of the anal region in CD [39] and is highly useful for the detection and definitive diagnosis of rectal and anal cancers [40]. By combining this with endoscopic examination, it is possible to simultaneously assess the disease activity of CD and perform surveillance. Patients must fully understand the need for surveillance to continue with it. When endoscopic surveillance is combined with EUA, the rate of cancer detection in patients with CD is 5.8%, which may contribute to early diagnosis [41].

Currently, early diagnosis can be achieved by performing regular and continuous surveillance based on colonoscopy and EUA combined with MRI, CT, and tumor marker assessment, as appropriate. In high-risk patients, such as those with worsening local symptoms or long-term cases, repeated surveillance at short intervals is required even if no malignant findings are detected, while always suspecting the possibility of cancer.

## 8. Conclusions and Future Directions

As there are regional differences in the common sites and histological types of CD-related cancers, each region requires its own surveillance and treatment strategies. In particular, CD-related rectal and anal cancers have a poor prognosis, with a high incidence of local recurrence compared to that of sporadic colorectal cancers. As it is necessary to aim for an early diagnosis, regular surveillance must be performed while always suspecting the possibility of cancer. In addition, surgery to ensure the safety surgical margin is essential for local control of rectal cancer, and in the future, we believe that thorough R0 resection, which does not retain any cancerous tissue on the dissected surface, combined with preoperative CRT, will be key to improving prognosis.

## Abbreviations

The following abbreviations are used in this manuscript:

CD	Crohn’s disease
FDG-PET	Fluorodeoxyglucose positron emission tomography
EUA	Examination under anesthesia
